# How taphonomic alteration affects the detection and imaging of striations in stab wounds

**DOI:** 10.1007/s00414-017-1715-2

**Published:** 2017-10-29

**Authors:** Sophie A. Stanley, Sarah V. Hainsworth, Guy N. Rutty

**Affiliations:** 1 College of Medicine, Biological Sciences and Psychology, University of Leicester, Leicester, UK; 2 School of Engineering and Applied Science, Aston University, Aston, Birmingham, UK; 3 East Midlands Forensic Pathology Unit, University of Leicester, Robert Kilpatrick Building, Leicester Royal Infirmary, Leicester, UK

**Keywords:** Forensic imaging, Stabbing, Taphonomy, Micro-computed tomography, Striations, Serrated blade

## Abstract

Stabbing with a kitchen knife is a common method of homicide in Europe. Serrated knives may leave tool markings (striations) in tissues. Documentation of striations is necessary for their use as forensic evidence. Traditional methods (physical casting and photography) have significant limitations, and micro-computed tomography (micro-CT) has been trialled in cartilage to “virtually cast” wounds. Previous research has shown the proportion of striations in cartilage falls following decomposition. This project has investigated the effects of taphonomic alteration and documentation methods of striations in porcine skin. Fresh, decomposed, mummified, burnt and waterlogged stab wounds in a porcine analogue were excised and imaged using photography, stereo-optical microscopy and micro-CT. The proportion of striations in each taphonomic group was determined from the images by independent analysts. Striations were observed more frequently in serrated blade wounds, although they were also identified in non-serrated blade wounds. The proportion of wounds showing striations declined following decomposition. An inversely proportional linear correlation between advancing decomposition and proportion of striations existed. Dehydration (mummification and burning) rendered serrated and non-serrated blade wounds indistinguishable. Water composition affected the preservation of striations. Identification of striations gradually declined after decomposition in tap water, but persisted to a point when left in brackish water. All three techniques imaged striations; however, the optimum technique was stereo-optical microscopy due to practical advantages and specific limitations affecting photography and micro-CT. This study demonstrates the effects of taphonomic alteration on striations and suggests stereo-optical microscopy is the optimum method for their documentation.

## Introduction

Stabbing is the most common method of homicide in the UK and much of Europe with 36% of cases in the UK in 2014/2015 involving a sharp implement [1–4]. Kitchen knives are commonly used as a weapon and may have features such as serrations [2]. When a serrated blade is used to stab tissue, individual serration points may leave markings in the tissue known as striations.

Striations were first recognised by Bonte et al. [5] in cases of thoracic stabbing and have since been observed experimentally in human and animal soft tissues, including skin [6–8]. Since the National Academy of Sciences report stating a requirement for forensic methodology to be quantitatively validated and peer reviewed, there has been limited investigation into the reliability of striations for weapon identification [9]. In cartilage, blade misclassification rates using striations have been found to be 4–21 and 50–66% in two different studies [10, 11]. The optimum method for the documentation of striations is also unknown. Crowder et al. [10] established similar error rates when comparing direct and indirect (casting) observation and digital and light microscopy in cartilage wounds. Establishing the optimum method for documentation, particularly in soft tissues, has been challenging. Casting in dental impression material was first suggested by Rao et al. [12]; however, limitations in this technique such as degradation of the original sample and inaccurate representations of striations due to poor material adherence, tissue shrinkage or intrinsic tissue features imprinting preferentially have meant that alternative methods have been investigated. Pounder et al. [13] demonstrated “virtual casting” using micro-CT and generated a three-dimensional (3D) model of a cartilage stab wound with visible striations. Other techniques such as stereo-optical microscopy and photography can also image striations, although tissue reflections hinder these methods [6–8, 10, 11, 14–16]. Lastly, the majority of previous striation research has been undertaken in fresh tissue. Most recently, Spagnoli et al. [14] have shown that the proportion of striations observed in cartilage decreases after 1 week of decomposition.

This research has shown that striations persist in porcine skin following taphonomic alteration, the extent of which is dependent on the taphonomic process and its duration. Stereo-optical microscopy, photography and micro-CT were all found to be capable of imaging striations. Of these, stereo-optical microscopy was found to be the best technique owing to its practical ease and availability and the potential limitations affecting photography and micro-CT.

## Method

Seven different sections of pork belly were purchased from a local butcher for use in this study. Tissue sections comprised skin, subcutaneous fat and muscle. Ribs were removed from the sections by the butcher as stabbing bone in a pilot trial made it difficult to remove the knife from the wound and often caused damage to the knife tips. Ribs would also have introduced variation between the wounds depending on whether the stab wound was inflicted over bone or not. Animals were typically 6–10 months old at slaughter which occurred 5–7 days before the meat was purchased. Care was taken to ensure the skin was not scored in the butchering process.

**Fig. 1:**
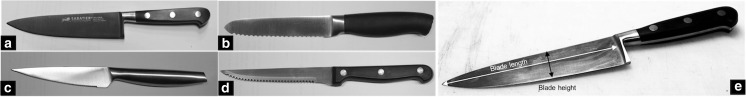
Knives used to stab porcine tissue demonstrating a range of different characteristics. Knife names correspond to data in Table 1. Knife manufacturers are **a** Sabatier, France, **b** OXO, USA, **c** John Lewis, UK, and **d** Chef’s Own, UK. **e** Illustration of the measurements taken on the knives that are listed in Table 1

Tissue sections were stabbed on the day of purchase in a room typically 20 °C using four kitchen knives purchased from the UK high street. Three knives were serrated, and one knife had a plain edge blade. The characteristics of each knife and their manufacturers are shown in Table 1 and Fig. 1. Three wounds were inflicted per knife for each of the taphonomic categories.

**Table 1:** Characteristics of the knives used in this study

Knife	Use	Grind	Serration	Point	Blade length (mm)	Blade height (mm)	Weight (g)	Inter-serration distance (mm)	Side scalloped	Weight (g)	Drop height (m)
A	Chef	Taper	None	Drop	148	32.6	120	–	–	120	1.78
B	Utility	Taper	Coarse	Drop	123	20.7	82	4.64	Right	82	1.86
C	Utility	Taper	Fine	Straight	115	24.5	68	1.33	Left	68	1.89
D	Steak	Hollow	Coarse	Straight	111	15.2	24	3.23	Left	24	2.04

**Fig. 2:**
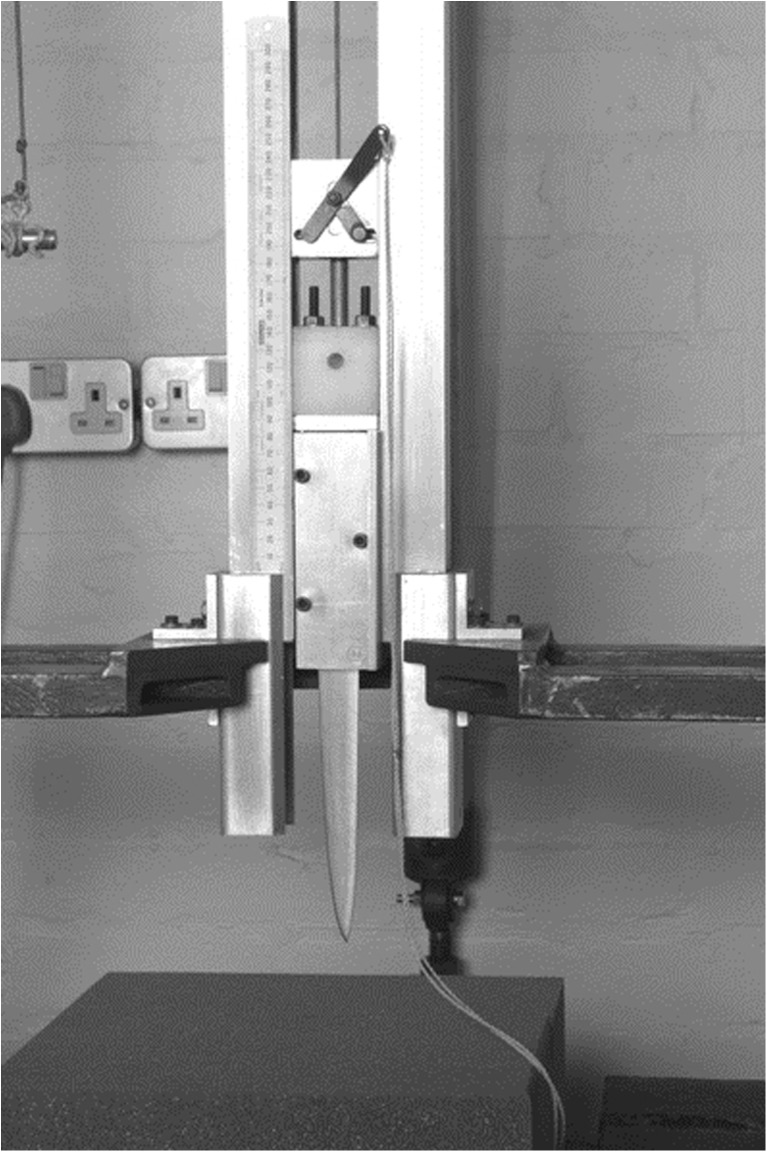
Drop tower that was used to create stab wounds in tissue sections. Tissue was placed beneath the knife point which was raised to a set height and released onto the tissue creating a stab wound. Image reproduced with the kind permission of Hainsworth et al. [17]

Knife names correspond with Fig. 1. Knife blade terminology used is defined by Pounder et al. [15, 16]. Knife blade measurements are detailed in Fig. 1. Sides of the knife are defined as the sides according to the person holding the knife e.g. the left side is scalloped if the blade to the left side of the person holding the knife, by the handle, is ground. Drop height refers to the height from which each knife was dropped using the drop tower (Fig. 2) to ensure stab wounds were inflicted with a constant kinetic energy of 15 J

Figure 2 shows the drop tower method used to stab tissue. This has been used in previous stabbing research, as it allows the force and direction of the wound to be controlled [17, 18]. The kinetic energy used to stab the tissue was kept constant at 15 J meaning that the drop heights were altered to compensate for the different weight knives (Table 1). The weight of the drop tower carriage (approx. 738 g) was also accounted for when calculating the drop height. Knife tips were aligned perpendicularly to the skin before being dropped onto the tissue to create a stab wound. After stabbing, the knives were removed from the tissue manually with care taken to remove them at the same angle at which they entered the tissue with minimal lateral movement. Knife blades were wiped clean between each stab wound and checked for visible damage. No knives were damaged during stabbing, therefore no replacement knives were used. Multiple stab wounds (16–36 per section) were inflicted into each section of tissue.

After all the stab wounds had been inflicted into a section, they were immediately transferred to the environmental conditions intended to alter the tissue, except for the fresh samples. Fresh stab wounds were immediately excised.

To produce the decomposed, mummified and waterlogged samples, sections of stabbed tissue were left in an incubator (Memmert, Germany) at 25–31 °C and 37–41% humidity. The temperature and humidity data was recorded continuously throughout the experiment using a remote datalogger (SensorMetrix, UK).

The time that each sample was left differed for the different taphonomic groups, but all were measured in accumulated degree days (ADDs). ADDs represent the cumulative thermal energy available to a biological or chemical process [19, 20]. They eliminate the temperature variable which is shown to most significantly affect the rate of decomposition, allowing comparison between other taphonomic studies in different conditions [20]. To calculate ADDs, a base temperature is set (0 °C) at which the process is inhibited, and average daily temperatures are totalled over a given period of time (days) [19, 21]. Post-mortem intervals can be estimated from the observable features of decomposition (both terrestrial and aquatic) due to a predictive formula that relates total body score (or total aquatic decomposition score) and ADDs [19, 22, 23]. The durations and specific conditions for each incubated sample are shown in Table 2.

**Table 2:** Stabbed sections of tissue were left in specific conditions, detailed in this table, intended to replicate taphonomic processes e.g. mummification, terrestrial and aquatic decomposition

Sample	Conditions	Samples removed (ADD)			
Terrestrial decomposition	Sealed plastic container to prevent tissue dehydration	71	150	208	266
Mummified	Open plastic container allowing dehydration	591			
Aquatic decomposition	Immersed in tap water in a sealed plastic container	71		150	
	Immersed in brackish water in a sealed plastic container	68		133	

Individual stab wounds were removed for imaging at different time intervals measured using accumulated degree days (ADDs)

For the waterlogged samples, tap water was obtained from the Robert Kilpatrick Clinical Sciences Building at the Leicester Royal Infirmary, UK, on the day of immersion. Brackish water was obtained from the lock gate area at Boston Docks, Lincolnshire, UK. It was collected at half tide (ebbing) and transported to the Leicester Royal Infirmary in a plastic container. It was stored at room temperature for 6 days before use in this experiment. The osmolality of both water samples was measured and was 6 and 215 mOsm kg^−1^ for the tap and brackish water respectively.

Tissue was burnt in an air recirculating chamber furnace (Lenton, UK) at 395–407 °C for 11 min. The furnace was heated to temperature prior to the tissue being placed inside. Tissue was observed at 5, 7, 9 and 11 min. Tissue was removed when skin was charred and blistered but not completely cremated. The tissue was removed from the furnace and cooled to room temperature before excision of the wounds.

Stab wounds were excised from larger sections of tissue by cutting around the stab wound with a scalpel and separating the skin from underlying subcutaneous tissue by blunt dissection or using scissors. Care was taken to leave a sufficient margin around the stab wound. The mean dimensions of excised wounds were 37 × 17 × 4 mm. For incubated samples, individual wounds were excised and the parent tissue section returned to the incubator if remaining wounds required further decomposition. Wounds were opened by extending the stab track to the edge of the excised sample with a scalpel so that both stab track walls could be visualised. Care was taken not to touch the track walls in this process so that any markings were not disturbed or artificially created.

Samples were photographed immediately after opening with a Canon EOS 600D digital camera (Canon, Japan) with a 100-mm macro lens positioned on a tripod over a white background. Photographs were taken at different angles so that striations could be optimally visualised. An Olympus SZX12 stereo-optical microscope (Olympus, Japan) with an Olympus DP70 digital camera was then used to image the same sample. This was done within 8 h of excision. Samples were imaged with the lens of the microscope positioned perpendicularly over the sample.

After imaging with stereo-optical microscopy, the stab wounds were preserved for 6 days in 10% formalin solution. Samples were removed from the formalin solution, washed thoroughly under running water and dried with paper towel. The preserved samples were stored in air-tight containers until imaging with micro-CT. The wound tracks were stained with iodine prior to imaging to assist assessment. A Nikon Metrology XT H 225 (Nikon, Japan) micro-CT scanner was used to image the samples using a voltage of 53 kV, with a current of 1132 μA and exposure times per radiograph of 500 ms. Images were taken every 0.1° resulting in 3016 radiographs which were reconstructed using CT-Pro software (Nikon, Japan) as a .vgl file. This was visualised as a 3D model, analysed and edited using VGStudio MAX (Volume Graphics, Germany).

As stab wounds were inflicted and imaged by the same individual (SAS), four independent medical student observers were used to analyse images of the samples on an Apple LED cinema display LCD monitor with 27″ screen, 2560 × 1440 resolution (Apple, USA). Observers received identical training prior to analysis, and image sizes remained constant so that they could be viewed at 100% in accordance with the screen resolution. Observers were blinded to the knife used to inflict each wound and to each other’s answers. They were asked to state whether an image showed striations or not.

Observer responses were used to calculate the proportion of striations observed in each taphonomic and imaging group. The proportion of striations identified in each group was compared using a chi-squared test for independence. Yates’ correction was applied. Pearson’s product-moment correlation coefficient (PPMCC) was calculated for decomposed samples to assess the relationship between ADDs and the proportion of striations observed. All statistical calculations were performed using software R version 3.2.4 (R Foundation for Statistical Computing, Austria).

## Results

Four observers analysed 790 images of 132 stab wounds. In each of the 11 categories (e.g. fresh, burnt), the four knives inflicted three wounds each giving a total of 12 wounds. These 12 wounds were imaged twice by each of the three techniques generating 72 images per category.^1^ Two randomly allocated observers (all four in the fresh group) then interpreted the images, and their responses (*n*) were used to calculate the proportion of visible striations. For example, three wounds from knife A in the fresh group would generate six photographs which would be analysed by all four observers generating 24 responses i.e. *n* = 24 as quoted in Tables 3, 4, 6 and 7.

**Table 3:** The proportion of wounds where observers identified striations in both plain edge (A) and serrated blade (B, C, D) wounds imaged using photography, stereo-optical microscopy (SOM) and micro-CT in fresh tissue

Non-serrated blade	Proportion of wounds showing striations (%)			Serrated blades	Proportion of wounds showing striations (%)		
	Photography	SOM	Micro-CT		Photography	SOM	Micro-CT
A_(*n* = 24)_	41.7	29.2	33.3	B _*n* = 24)_	37.5	75.0	41.7
				C_(*n* = 24)_	95.9	100	12.5
				D_(*n* = 24)_	54.2	70.8	91.7

**Table 4:** The proportion of wounds where observers identified striations in both plain edge (A) and serrated blade (B, C, D) wounds imaged using photography, stereo-optical microscopy (SOM) and micro-CT in tissue that had been left to decompose in air for different durations as measured using accumulated degree days (ADDs)

Non-serrated blade	Proportion of wounds showing striations at different ADD intervals (%)					Serrated blades	Proportion of wounds showing striations at different ADD intervals (%)				
	0^a^	71	150	208	266		0^a^	71	150	208	266
Photography											
A_(*n* = 12)_	41.7	66.7	58.3	66.7	41.7	B_(*n* = 12)_	37.5	41.7	75.0	75.0	33.3
						C_(*n* = 12)_	95.9	66.7	66.7	66.7	66.7
						D_(*n* = 12)_	54.2	66.7	66.7	58.3	58.3
SOM											
A_(*n* = 12)_	29.2	33.3	41.7	25.0	25.0	B_(*n* = 12)_	75.0	66.7	16.7	58.3	10.0^b^
						C_(*n* = 12)_	100	50.0	33.3	50.0	41.7
						D_(*n* = 12)_	70.8	50.0	41.7	33.3	8.33
Micro-CT											
A_(*n* = 12)_	33.3	33.3	66.7	8.33	33.3	B_(*n* = 12)_	41.7	41.7	41.7	41.7	40.0^b^
						C_(*n* = 12)_	12.5	33.3	58.3	25.0	58.3
						D_(*n* = 12)_	91.7	41.7	41.7	25.0	8.33

^a^ *n* = 24

^b^ *n* = 10

Table 3 shows the proportion of striations seen in fresh stab wounds i.e. at 0 ADDs. Striations were observed in both non-serrated and serrated blade wounds. There is variation between the proportion of striations observed depending on the knife and the technique used to image the wound.

Every stab track wall was imaged using all three techniques; therefore, theoretically, any variation in the proportion of striations identified by different imaging techniques is because of the techniques themselves. Only fresh results (Table 3) demonstrated a significant difference between the proportion of striations identified by different imaging techniques. Micro-CT showed significantly fewer striations in knife C wounds compared to photography (*n* = 24, *χ* ^2^ = 30.3, *p* = 3.71 × 10^−8^) and stereo-optical microscopy (*n* = 24, *χ* ^2^ = 33.8, *p* = 5.92 × 10^−9^). This suggests that the micro-CT knife C result is anomalous and due to differences in imaging rather than a representation of the true proportion of visible striations. Excluding this value, a higher proportion of serrated (70.9%) as opposed to plain edge (34.7%) bladed wounds shows striations.

**Fig. 3:**
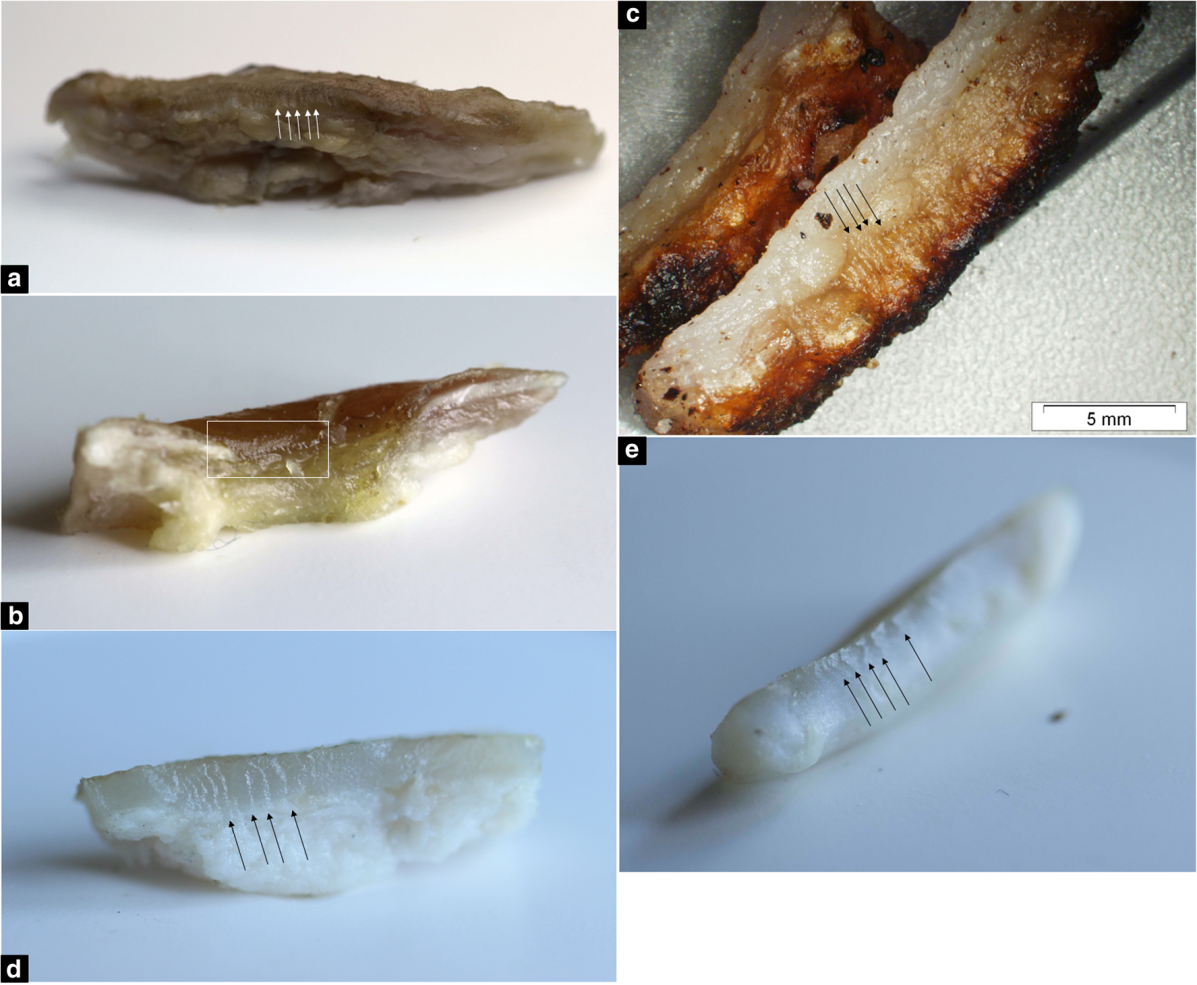
Photographic images (except **c** stereo-optical microscopy) in which all observers identified striations (arrows and box). **a** Knife C wounds at 208 ADDs terrestrial decomposition. **b** Mummified knife B wound. **c** Burnt knife C wound. **d** Knife D wound at 68 ADDs in brackish water. **e** Knife B wound at 71 ADDs in tap water

It was possible to image some striations in all taphonomic groups (Fig. 3). The consistency of this finding was affected by the knife used, imaging technique and taphonomic process affecting the tissue.

**Fig. 4:**
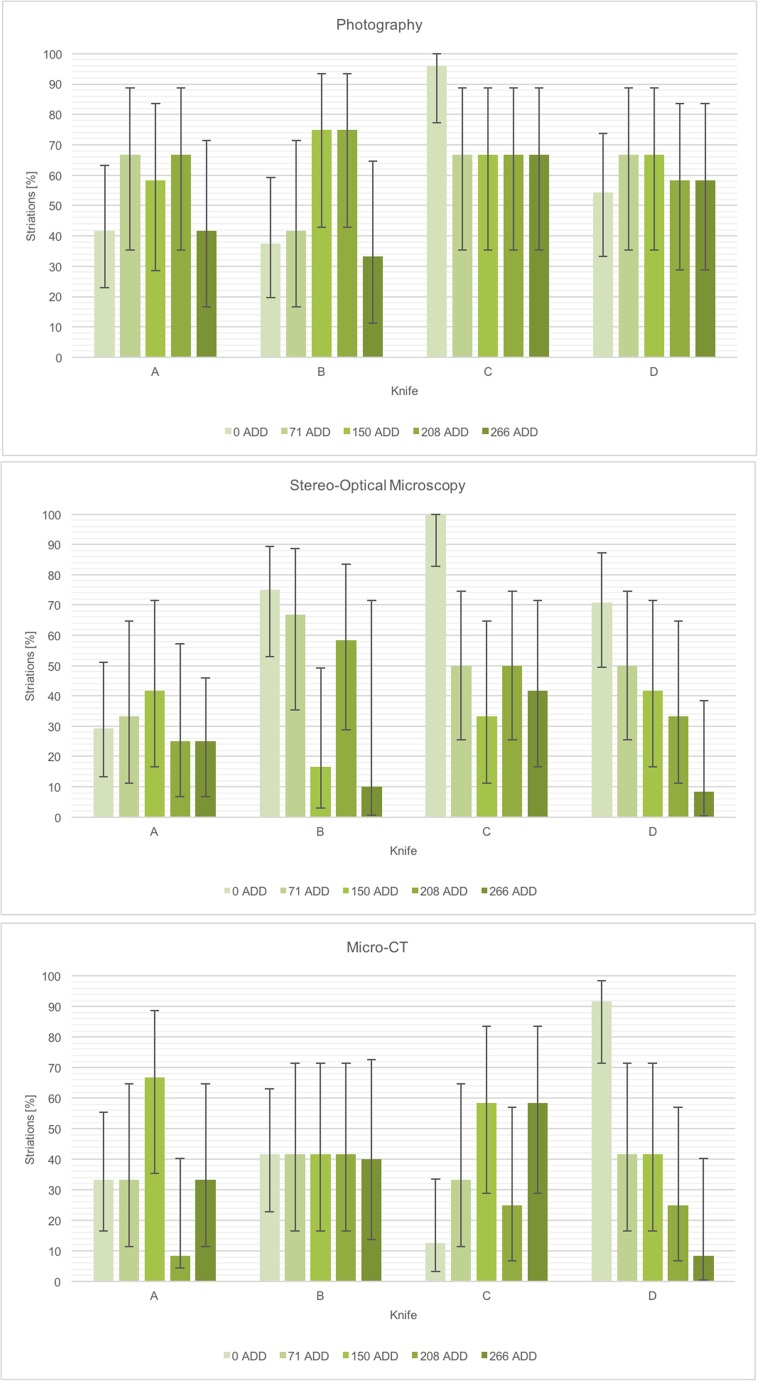
The proportion of striations identified in plain edge (knife A) and serrated (knives B, C and D) blade stab wounds after different periods of decomposition as measured by accumulated degree days (ADDs). The same wounds were imaged using photography, stereo-optical microscopy and micro-CT, and the proportions identified from images produced using each technique are shown in the three graphs. The error bars represent the 95% confidence intervals for each proportion as calculated using a chi-squared approximation with Yates correction applied

The proportion of wounds showing striations at different ADD intervals is shown in Table 4 and Fig. 4.

- In plain edge bladed wounds (A), the proportion of striations in fresh wounds (0 ADD) did not differ significantly from the proportions seen at 71, 150, 208 or 266 ADDs, regardless of imaging technique used.
- In wounds from serrated blades, the proportion of striations in fresh wounds (0 ADD) did not differ significantly from the proportions seen at 71, 150, 208 or 266 ADDs, when imaged using photography.
- In wounds from serrated knife C, imaged using stereo-optical microscopy (SOM), the proportion of striations in fresh wounds (0 ADD, *n* = 24) was significantly different to the proportions observed at 71 (*n* = 12, *χ* ^2^ = 11.0, *p* = 0.000899), 150 (*n* = 12, *χ* ^2^ = 16.9, *p* = 3.95 × 10^−5^), 208 (*n* = 12, *χ* ^2^ = 11.0, *p* = 0.000899) and 266 (*n* = 12, *χ* ^2^ = 13.9, *p* = 0.000198) ADDs i.e. after any period of decomposition.
- In wounds from serrated knife D, imaged using SOM, the proportion of striations in fresh wounds (0 ADD, *n* = 24) was significantly different to the proportion observed at 266 (*n* = 12, *χ* ^2^ = 10.1, *p* = 0.00146) ADDs i.e. after the longest period of decomposition.
- In wounds from serrated knife D, imaged using micro-CT, the proportion of striations in fresh wounds (0 ADD, *n* = 24) was significantly different to the proportions observed at 71 (*n* = 12, *χ* ^2^ = 8.17, *p* = 0.00427), 150 (*n* = 12, *χ* ^2^ = 8.17, *p* = 0.00427), 208 (*n* = 12, *χ* ^2^ = 13.8, *p* = 0.000208) and 266 (*n* = 12, *χ* ^2^ = 20.6, *p* = 5.65 × 10^−6^) ADDs i.e. after any length of decomposition.

Testing for a linear correlation between the proportion of striations identified and ADDs was performed (Table 5). A significant inversely proportional linear relationship was found when serrated knife D wounds were imaged with SOM (*n* = 5, *R* = −0.97, *p* = 0.006) and with micro-CT (*n* = 5, *R* = −0.93, *p* = 0.021).

**Table 5:** Pearson’s product-moment correlation coefficient (*R*) indicating the relationship between decomposition as measured using accumulated degree days (ADDs) and the proportion of striations visible in wounds made using plain edge (A) and serrated (B, C, D) blades

Knife	Photography		Stereo-optical Microscopy		Micro-CT	
	R	*p*	R	*p*	R	*p*
A_(*n* = 5)_	0.04	0.948	− 0.33	0.593	− 0.15	0.805
B_(*n* = 5)_	0.25	0.688	− 0.41	0.495	− 0.67	0.216
C_(*n* = 5)_	−0.73	0.159	− 0.74	0.153	− 0.67	0.219
D_(*n* = 5)_	0.04	0.951	*− 0.97*	*0.006*	*− 0.93*	*0.021*

An *R* value of 0 indicates no relationship between the variables. An *R* value of − 1 indicates and perfectly linear inversely proportional relationship i.e. as one variable increases, the other decreases at the same rate. The *p* value for each coefficient is given. Only *p* values < 0.05 were considered significant (italics)

Table 6 shows the proportion of striations seen in fresh, mummified and burnt wounds. The proportion of striations observed in fresh plain edge bladed (A) and mummified plain edge bladed (A) wounds did not differ significantly. No significant difference was seen between fresh and mummified serrated blade wounds imaged by photography. A significant difference was seen between fresh (*n* = 24) and mummified knife C wounds imaged by SOM (*n* = 12, *χ* ^2^ = 11.0, *p* = 0.000899) and knife D wounds imaged with micro-CT (*n* = 12, *χ* ^2^ = 8.17, *p* = 0.00427). There is no consistent trend in the identifiability of striations following mummification with approximately half the categories (5/12) showing an increase and half showing a decrease (6/12) in the proportion of striations observed. The proportion of striations in burnt wounds never differed significantly from the proportion found in fresh wounds. After burning, a consistent trend (i.e. increase or decrease) could not be identified and the proportion in plain edge blade (58.3%) and serrated blade (50.0–66.7%) wounds became very similar (Table 6).

**Table 6:** The proportion of wounds where observers identified striations in both plain edge (A) and serrated blade (B, C, D) wounds imaged using photography, stereo-optical microscopy (SOM) and micro-CT in fresh, mummified and burnt tissue

Non-serrated blade	Proportion of wounds showing striations (%)			Serrated Blades	Proportion of wounds showing striations (%)		
	Fresh^a^	Mummified	Bumt		Fresh^a^	Mummified	Bumt
Photography							
A_(*n* = 12)_	41.7	66.7	58.3	B_(*n* = 12)_	37.5	83.3	50.0
				C_(*n* = 12)_	95.9	58.3	58.3
				D_(*n* = 12)_	54.2	91.7	50.0
SOM							
A_(*n* = 12)_	29.2	75.0	58.3	B_(*n* = 12)_	75.0	41.7	66.7
				C_(*n* = 12)_	100	50.0	66.7
				D_(*n* = 12)_	70.8	58.3	50.0
Micro-CT							
A_(*n* = 12)_	33.3	25.0	58.3	B_(*n* = 12)_	41.7	41.7	58.3
				C_(*n* = 12)_	12.5	66.7	58.3
				D_(*n* = 12)_	91.7	41.7	58.3

^a^ *n* = 24

**Fig. 5:**
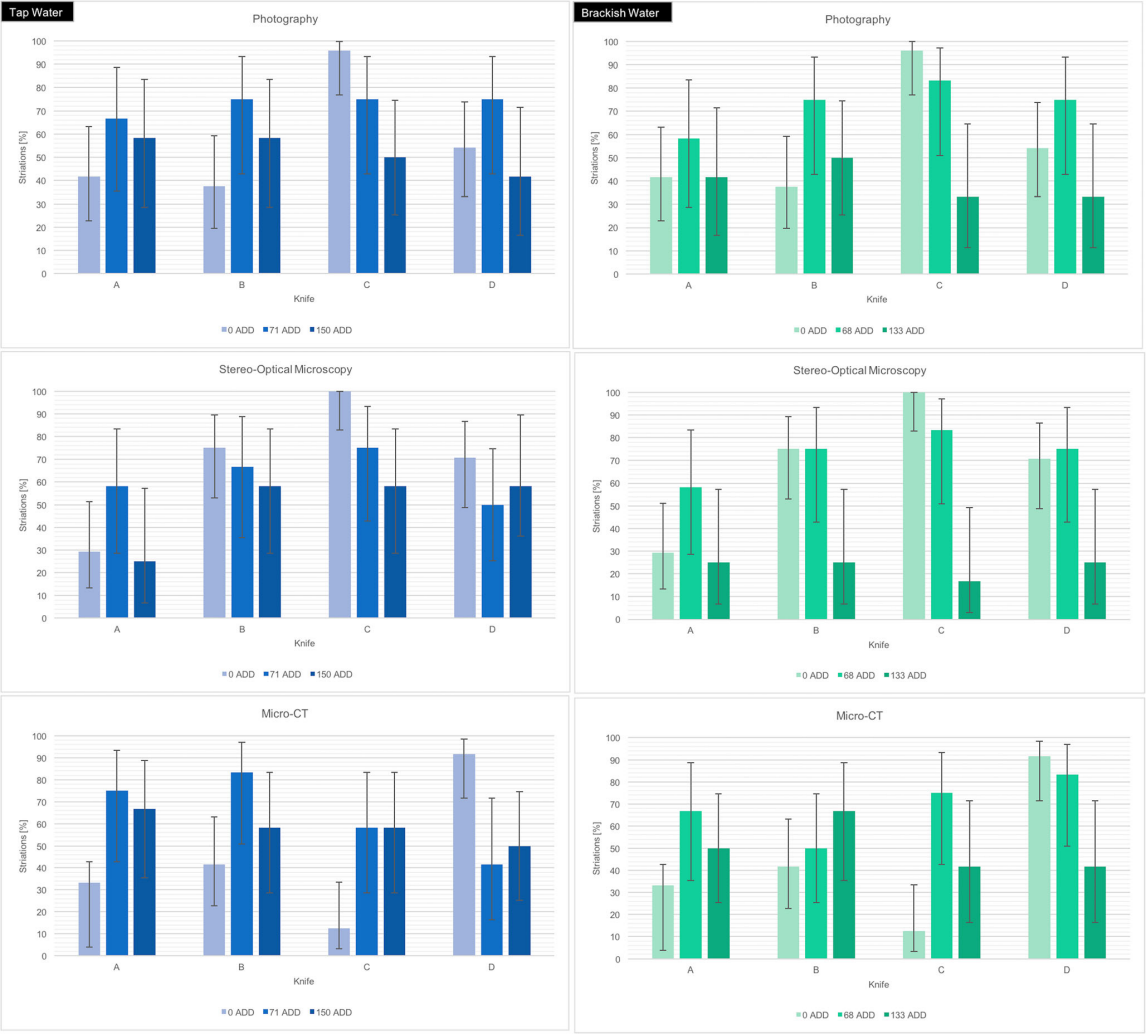
The proportion of striations identified in plain edge (knife A) and serrated (knives B, C and D) blade stab wounds after different periods of decomposition in tap (left column) and brackish (right column) water as measured by accumulated degree days (ADDs). The same wounds were imaged using photography, stereo-optical microscopy and micro-CT, and the proportions identified from images produced using each technique are shown in the three graphs. The error bars represent the 95% confidence intervals for each proportion as calculated using a chi-squared approximation with Yates correction applied

Table 7 and Fig. 5 show the proportion of striations observed in fresh wounds and in wounds left in tap water or brackish water.

- In plain edge bladed wounds (A), the proportion of striations in fresh wounds (0 ADD) did not differ significantly from the proportions seen after immersion in both tap and brackish water for any length of time when any imaging technique was used.
- In wounds from serrated knife C imaged using photography, the proportion of striations in fresh wounds (0 ADD, *n* = 24) was significantly different to the proportion observed at 150 ADD in tap water (*n* = 12, *χ* ^2^ = 8.00, *p* = 0.00467) and 133 ADD in brackish water (*n* = 12, *χ* ^2^ = 13.5, *p* = 0.000239).
- Serrated knife C wounds imaged using SOM also showed a significantly different proportion of striations identified after 133 ADD in brackish water (*n* = 12, *χ* ^2^ = 23.7, *p* = 1.13 × 10^−6^) when compared with fresh wounds (0 ADD, *n* = 24).
- In wounds from serrated knife D, imaged with micro-CT, there was a significant difference in the proportion of striations shown in fresh wounds (0 ADD, *n* = 24) and those left in tap water for 71 ADDs (*n* = 12, *χ* ^2^ = 8.17, *p* = 0.00427) and left in brackish water for 133 ADDs (*n* = 12, *χ* ^2^ = 8.17, *p* = 0.00427).

**Table 7:** The proportion of wounds where observers identified striations in both plain edge (A) and serrated blade (B, C, D) wounds imaged using photography, stereo-optical microscopy (SOM) and micro-CT in tissue that had been left to decompose in tap and brackish water for different durations as measured using accumulated degree days (ADDs)

Non-serrated blade	Proportion of wounds showing striations at different ADD intervals (%)					Serrated blades	Proportion of wounds showing striations at different ADD intervals (%)				
		Tap water		Brackish water				Tap water		Brackish water	
	0^a^	71	150	68	133		0^a^	71	150	68	133
Photography											
A_(*n* = 12)_	41.7	66.7	58.3	58.3	41.7	B_(*n* = 12)_	37.5	75.0	58.3	75.0	50.0
						C_(*n* = 12)_	95.9	75.0	58.3	83.3	33.3
						D_(*n* = 12)_	54.2	75.0	41.7	75.0	33.3
SOM											
A_(*n* = 12)_	29.2	58.3	25.0	58.3	25.0	B_(*n* = 12)_	75.0	66.7	58.3	75.0	25.0
						C_(*n* = 12)_	100	75.0	58.3	83.3	16.7
						D_(*n* = 12)_	70.8	50.0	58.3	75.0	25.0
Micro-CT											
A_(*n* = 12)_	33.3	75.0	66.7	66.7	50.0	B_(*n* = 12)_	41.7	83.3	58.3	50.0	66.7
						C_(*n* = 12)_	12.5	58.3	58.3	75.0	41.7
						D_(*n* = 12)_	91.7	41.7	50.0	83.3	41.7

^a^ *n* = 24

Generally, the proportion of striations observed in serrated blade wounds declines gradually with time left in tap water. This is illustrated by the average (mean) proportion of wounds showing striations which declines from 70.9%^2^ to 66.7 to 55.5% at 0, 71 and 150 ADDs respectively. The changing proportion seen in plain edge bladed wounds does not show a gradual decline (0 ADD = 34.7%, 71 ADDs = 66.7%, 150 ADDs = 50.0%), but does show similar absolute values at the later ADD intervals after immersion in tap water. Overall, the proportion of striations observed in serrated blade wounds left in brackish water at 68 ADDs (mean = 74.9%) is similar to that seen at 0 ADD (mean = 70.9%). However, by 133 ADDs there is a reduction in the proportion seen in serrated blade wounds to 37.0% (mean). The proportion in plain edge bladed wounds fluctuates with time left in brackish water (0 ADD = 34.7%, 68 ADDs = 61.1%, 133 ADDs = 38.9%) although similar absolute proportions are seen in serrated and non-serrated blade wounds at 133 ADDs.

**Fig. 6:**
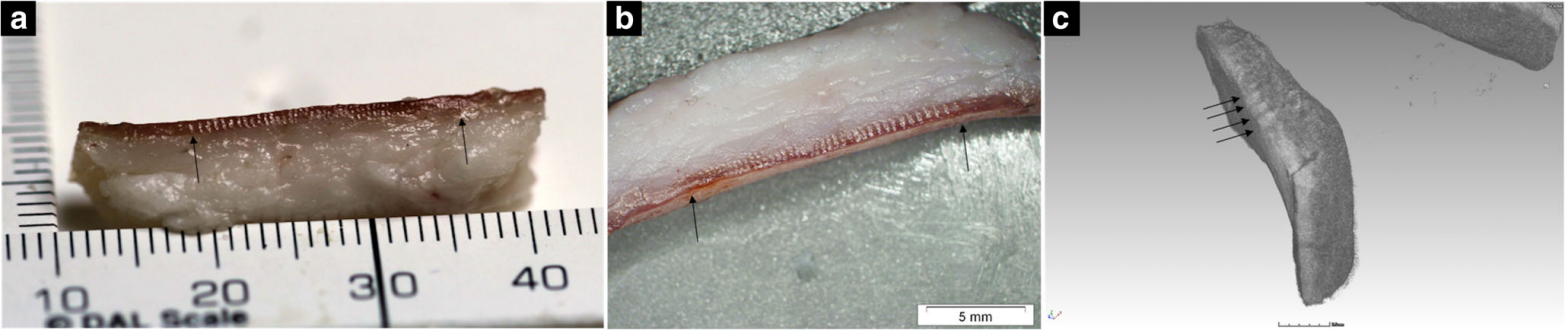
Striations (indicated by arrows) could be imaged in fresh wounds made using serrated knives with **a** photography (knife C), **b** stereo-optical microscopy (knife C) and **c** micro-CT (knife D)

All imaging techniques were capable of imaging striations (Fig. 6). Significant differences between the striation rate identified in the same wounds were only found in the fresh data set (Table 3). In the fresh data set, SOM demonstrated the greatest difference between the proportion of striations imaged in plain edge bladed (29.2%) and serrated bladed wounds (mean = 81.9%). SOM also shows the least variation (range = 29.2%) in the striation rates found for the serrated blades compared to photography and micro-CT, range = 58.2 and 79.2% respectively. In all other taphonomic categories, there were no significant differences between the imaging techniques in the proportion of striations identified.

## Discussion

As previous research has found, striations are not exclusive to serrated blade wounds as non-serrated blades also produce markings. Potential explanations for this include knife blade imperfections e.g. from grinding or usage; knife blade vibration as it enters or leaves the tissue; or biological variation in tissue samples mimicking wound markings. The proportion of plain edge bladed wounds showing striations in this work is consistent with that of other studies; however, it is a significant false-positive result that limits the transferability of this work to forensic practice [14]. To minimise the identification of striations in plain edge bladed wounds, Spagnoli et al. [14] suggest asking observers to identify a striation pattern rather than just the presence of striations. Crowder et al. [10] suggest that greater accuracy in striation identification may be achieved by using tool mark examiners with greater experience. A limitation of this study may therefore be the use of medical students as observers who were inexperienced in tool mark analysis. Studies such as this frequently quote inter-observer reliability statistics. However, given the effect of prevalence on kappa statistics as outlined by Feinstein et al. [24], and the small number of observer responses in the individual sub-groups, a kappa statistic would likely be skewed and show an inappropriate representation of observer variability in this study.

Based on the fresh results, this study suggests that using stereo-optical microscopy could also increase the ability to differentiate between markings in plain edge and serrated blade wounds. SOM showed the greatest difference in the proportion of striations observed between serrated and non-serrated blade wounds. Practically, SOM allows for the control of more imaging variables such as lighting, lens positioning and magnification. During photography, these variables, e.g. angled lens and lighting, may have augmented certain wound features that were not necessarily striations resulting in a higher rate of false-positive results (i.e. striations seen in plain edge bladed wounds).

In addition, as the proportion of wounds showing striations varied least between the different serrated knives when imaged using SOM, this indicates SOM may be more capable of imaging a serration pattern from knives with a variety of different serration profiles. This was a limitation noted in the use of micro-CT. Micro-CT detected a high proportion of striations in knife D wounds. The most likely explanation for this is because the micro-CT scanner settings were calibrated for this knife during a pilot study undertaken to establish the method. These settings were applied to other knives, but may not have been appropriate. Knife C had a much smaller inter-serration distance; therefore, the resolution may have been insufficient to detect striations and hence caused the significantly lower striation rate seen for this knife. Inappropriate micro-CT setting calibration may have prevented the true imaging potential of micro-CT from being recognised in this work. In addition, this study overlooks the potential benefit of producing a 3D virtual model of a stab wound by asking observers to interpret a 2D still of the model rather than allowing them to manipulate and explore the structure fully. However, there are practical limitations associated with micro-CT such as higher cost, limited availability and greater technical expertise required for use that are not associated with SOM or photography.

There is variation in the absolute striation rates according to the type of knife used; however, generally similar trends are observed across all the serrated blades. The absolute differences in the proportion of striations observed are likely to relate to the different serration profiles of the knives. Some previous research [6, 15] has suggested that more coarsely serrated knives will leave more visible striations whereas Crowder et al. [10] suggest the opposite. This study found that knives with a smaller inter-serration distance left more visible striations (knife C compared to knife B) although it hypothesised that serration depth may also have affected the visibility of striations alongside inter-point distance.

Evidence that striations can persist following taphonomic alteration is demonstrated by qualitative interpretation of the images in this study. However, quantitative data representing the proportion of wounds showing striations suggests that this finding is not consistent and that after some form of taphonomic alteration, the striation rate becomes similar to that seen in plain edge bladed wounds.

In decomposed tissue, there is an almost linear decline in the proportion of wounds showing striations as time to decompose increases suggesting that these markings degrade with time (measured in ADDs). Significantly lower proportions were generally found in much later ADD groups (> 208 ADD), suggesting that after a certain point in time striations are no longer recognisable as distinct from other wounds features e.g. as a result decomposition. Spagnoli et al. [14] observed a large but non-significant reduction in the proportion of striations in cartilage following 1 week of air drying (equivalent to 113–161 ADDs, given an air temperature of 17–23 °C). The similarity in time frames in both this study and that of Spagnoli et al. [14] could indicate the point at which striations become unidentifiable in wounds following decomposition.

A constant striation rate was observed in mummified and burnt tissues regardless of the type of knife used. This could suggest the persistence of wound markings following these processes; however, misinterpretation of tissue or imaging artefacts are a more likely suggestion, particularly considering that the observers were inexperienced in assessing wound markings and that the striation rate seen in both plain edge and altered wounds became very similar. Spagnoli et al. [14] commented on the appearance of “cracks” and “ripples” on decomposition and how this, in addition to tissue discolouration, could prevent the identification of striations. Burning and mummification alter tissues by dehydration. By reducing the water content of the tissue, this causes it to contract which may degrade any wound markings present, more significantly than if the tissue remains wet e.g. in the case of decomposition.

In wounds subject to aquatic decomposition, the composition of the water appears to affect the preservation of striations. The proportion of striations observed in wounds left in tap water declined in a similar manner to samples that decomposed in air i.e. a gradual reduction in the proportion of serrated blade wounds showing striations until the proportion observed in plain edge bladed wounds was reached. However, the proportion of striations observed after 68 ADDs in brackish water remained similar to the proportion seen in fresh wounds. The proportion then drops to the level seen in plain edge blade wounds by 133 ADDs. The initial preservation of striations may be explained by the brackish water having a higher salinity, which is noted to reduce the decomposition rate of biological material [25].

As with all stabbing and taphonomic research, this study is limited by its ability to recreate these events experimentally. Variables such as the force and direction of the stab wounds were controlled; however, a drop tower can only replicate two of either impact velocity, momentum or energy present in the action of stabbing [17]. It is also unlikely that a stab wound would be inflicted perpendicularly in a real scenario, and the effect of penetration angle on the appearance of striations is not known.

In this experiment, burning has been simulated in a furnace which burns tissue by radiant heat; however, direct heat is more commonly applied following homicide particularly with the use of accelerants [26]. These methods degrade tissue in different ways e.g. radiant heat by dehydration compared to direct heat which uses the tissue as fuel [27]. These differences could influence the preservation of striations.

Although the use of ADDs negates the effect of temperature on decomposition and allows comparison between other taphonomic studies, other major variables that affect decomposition processes such as scavenger and entomological activity have not been replicated and could affect the preservation of striations. In all taphonomic simulations, the sequelae of decomposition in a small tissue section may also differ from its progression in a full corpse.

Lastly, this work was conducted in porcine skin. Although accepted as an analogue for human skin, porcine skin is thicker and therefore may affect the retention of striations [7, 28]. Other studies have found that striations are preserved in both porcine and human skin meaning the results from this project may be transferrable to a human situation [6, 8].

## Conclusions

In conclusion, the persistence of striations following taphonomic alteration has been assessed through analysis of images of serrated and non-serrated blade stab wounds in porcine skin that were exposed to different environmental conditions. The imaging techniques used were photography, stereo-optical microscopy and micro-CT, and the efficacy of these was also compared. The findings are as follows:

- Striations were observed in all stabs for all samples made with all the different types of blades. Thus, serrations can be observed even in stabs made with plain edge blades. This is a significant false-positive result that limits the use of striations for serrated blade identification.
- Striations were observed more frequently in stabs made with serrated blades than in stabs made with plain edge blades.
- The proportion of striations identified in serrated blade wounds decreases following terrestrial decomposition to the level seen in stabs made with plain edged blades. There is a significant inversely proportional linear relationship between advancing decomposition (measured in accumulated degree days) and the proportion of striations identified.
- The proportion of striations identified in serrated and plain edge blade wounds becomes very similar after alteration by dehydration (mummification and burning). There is no consistent trend (i.e. increase or decrease) in the proportion of striations identified following dehydration. This suggests serrated and plain edge blade wounds altered by dehydration become indistinguishable possibly due to the destruction of striations by tissue contraction and the creation of tissue artefacts.
- The proportion of striations seen in serrated blade wounds following immersion in tap water falls gradually to a level similar to that seen in flat bladed wounds. The proportion of striations identified in fresh samples and those left in brackish water remains similar until a point between 68 and 133 accumulated degree days when the proportion declines to the level seen in non-serrated blade wounds.
- Stereo-optical microscopy, photography and micro-CT were all found to be capable of imaging striations in all taphonomic categories. Stereo-optical microscopy was found to be the optimum technique as it produced images from which observers could most consistently distinguish between striations and markings from flat bladed knives. Stereo-optical microscopy holds practical advantages over micro-CT imaging owing to its ease of use, widespread availability and cost.

## Footnotes

1 Prior to imaging with stereo-optical microscopy and micro-CT, a Knife B sample from 266 ADD group was damaged therefore there are 790 as opposed to the 792 images expected.

2 The anomalous micro-CT Knife C result is excluded from this average

## References

[1] Flatley J (2016) Focus on violent crime and sexual offences: year ending march 2015. Office for National Statistics. https://www.ons.gov.uk/peoplepopulationandcommunity/crimeandjustice/compendium/focusonviolentcrimeandsexualoffences/yearendingmarch2015. Accessed 11 Feb 2016

[2] Karlsson T (1998). Homicidal and suicidal sharp force fatalities in Stockholm, Sweden. Orientation of entrance wounds in stabs gives information in the classification. Forensic Sci Int.

[3] Rogde S, Hougen HP, Poulsen K (2000). Homicide by sharp force in two Scandinavian capitals. Forensic Sci Int.

[4] Scolan V, Telmon N, Blanc A, Allery JP, Charlet D, Rouge D (2004). Homicide-suicide by stabbing study over 10 years in the Toulouse region. Am J Forensic Med Pathol.

[5] Bonte W (1975). Tool marks in bones and cartilage. J Forensic Sci.

[6] Jacques R, Kogon S, Shkrum M (2014). An experimental model of tool mark striations by a serrated blade in human soft tissues. Am J Forensic Med Pathol.

[7] Pounder DJ, Cormack L (2011). An experimental model of tool mark striations in soft tissues produced by serrated blades. Am J Forensic Med Pathol.

[8] Pounder DJ, Bhatt S, Cormack L, Hunt BA (2011). Tool mark striations in pig skin produced by stabs from a serrated blade. Am J Forenic Med Pathol.

[9] National Research Council (2009). Strengthening forensic science in the United States: a path forward.

[10] Crowder C, Rainwater CW, Fridie JS (2013). Microscopic analysis of sharp force trauma in bone and cartilage: a validation study. J Forensic Sci.

[11] Love JC, Derrick SM, Wiersema JM, Peters C (2012). Validation of tool mark analysis of cut costal cartilage. J. Forensic Sci.

[12] Rao VJ, Hart R (1983). Tool mark determination in cartilage of stabbing victim. J Forensic Sci.

[13] Pounder DJ, Sim LJ (2011). Virtual casting of stab wounds in cartilage using micro-computed tomography. Am J Forensic Med Pathol.

[14] Spagnoli L, Amadasi A, Frustaci M, Mazzarelli D, Porta D, Cattaneo C (2016). Characteristics and time-dependence of cut marks and blunt force fractures on costal cartilages: an experimental study. Forensic Sci Med Pathol.

[15] Pounder DJ, Cormack L, Broadbent E, Millar J (2011). Class characteristics of serrated knife stabs to cartilage. Am J Forensic Med Pathol.

[16] Pounder DJ, Reeder FD (2011). Striation patterns in serrated blade stabs to cartilage. Forensic Sci Int.

[17] Hainsworth SV, Delaney RJ, Rutty GN (2008). How sharp is sharp? Towards quantification of the sharpness and penetration ability of kitchen knives used in stabbings. Int J Legal Med.

[18] Horsfall I, Prosser PD, Watson CH, Champion SM (1999). An assessment of human performance in stabbing. Forensic Sci Int.

[19] Megyesi MS, Nawrocki SP, Haskell NH (2005). Using accumulated degree-days to estimate the postmortem interval from decomposed human remains. J Forensic Sci.

[20] Simmons T, Adlam RE, Moffatt C (2010). Debugging decomposition data—comparative taphonomic studies and the influence of insects and carcass size on decomposition rate. J Forensic Sci.

[21] Vass AA, Bass WM, Wolt JD, Foss JE, Ammons JT (1992). Time since death determinations of human cadavers using soil solution. J Forensic Sci.

[22] Heaton V, Lagden A, Moffatt C, Simmons T (2010). Predicting the postmortem submersion interval for human remains recovered from U.K. waterways. J Forensic Sci.

[23] Moffatt C, Simmons T, Lynch-Aird J (2016). An improved equation for TBS and ADD: establishing a reliable postmortem interval framework for casework and experimental studies. J Forensic Sci.

[24] Feinstein AR, Cicchetti DV (1990). High agreement but low kappa: I. The problems of two paradoxes. J Clin Epidemiol.

[25] Haglund WD, Sorg MH (1996). Forensic taphonomy: the postmortem fate of human remains.

[26] DeHaan JD, Icove DJ (2012). Kirk’s fire investigation.

[27] Fairgrieve SI (2008). Forensic cremation: recovery and analysis.

[28] Ankersen J, Birkbeck AE, Thomson RD, Vanezis P (1999). Puncture resistance and tensile strength of skin simulants. Proc Inst Mech Eng H.

